# Field experiment on the effect of musical street performance/busking on public space perception as mediated by street audience experience

**DOI:** 10.1038/s41598-024-62672-1

**Published:** 2024-06-07

**Authors:** Robbie Ho, Magdalena Szubielska

**Affiliations:** 1 Department of Cultural and Creative Arts, The Education University of Hong Kong, Hong Kong, Hong Kong; 2 Institute of Psychology, The John Paul II Catholic University of Lublin, Lublin, Poland

**Keywords:** Street performance, Busking, Public space, Environmental perception, Audience experience, Art appreciation, Psychology, Human behaviour

## Abstract

Street performance or busking is common in public spaces. The literature highlights two psychological issues: the effect of street performance on public space perception and the complexity of the appreciation of street performance, regarded as street audience experience (SAE). The present study aims at verifying the effect of street performance on public space perception, while examining SAE as a mediator of this effect. We conducted a between-subjects field experiment (a quasi-experiment; *N* = 292) in Hong Kong. Participants assessed a public space without (control) or with (experimental) musical busking on essentialism, anti-essentialism, sonic restorativeness, and overall liking. In the experimental condition, unengaged passersby and engaged audience further evaluated SAE factors of emotion, intellect, novelty, place, interaction, and technique, and outcomes of overall satisfaction and donation worth. The public space with busking was perceived as significantly more sonically restorative. Engaged audience perceived the space as significantly more essentialist, anti-essentialist, sonically restorative, and likeable. Engaged audience also experienced more positive SAE and outcome variables. SAE fully mediated the effects of street performance on public space perception and outcome variables, respectively. These findings support the positive impact of street performance, which may enhance city inhabitants’ well-being.

## Introduction

### Street performance

*Street performance*, or *busking*, refers to the act of performing or entertaining in a public space^1–3^. A *street performer*, or *busker*, is a person conducting a street performance. People passing by a street performance may or may not stop to spectate or watch the performance. Those who do not are referred to as *unengaged passersby*; those who do are referred to as *engaged audience*. Carrying on the ancient tradition of goliards, troubadours, minstrels, and the like, modern buskers seek to earn a living through street performance in exchange for voluntary tips or donations^4–7^.

To be open for anyone to watch without requiring a ticket at all^8^, street performance is typically conducted in public spaces. According to Carr et al.^9^, public spaces are “open, publicly accessible places where people go for group or individual activities” (p. 50). Also, according to Ehrenfeucht and Loukaitou-Sideris^10^, public spaces are “where people interact with those outside their private circles”, and the publicness of a space is “the extent to which people have access without permission, expressed or implied, and in which they can decide individually about how to conduct themselves” (p. 106). Furthermore, for street performers to maximize the number of potential audience, street performance is typically conducted in places with constant flows of pedestrian foot traffic, such as public squares or plazas, pedestrian streets, and transit stops^2,3,11^.

Street performance is common across cities around the world, spanning Australia, North and South Americas, UK, Europe^12^, and East Asia^13–15^. There are various types of street performance, and one broad classification is between musical (e.g., pop, jazz, classical) and nonmusical (e.g., juggling, miming, magic) performances^1–5,7,16,17^. While both types are representative of street performance, the present study focuses on the musical type, also regarded as street music.

### A psychological perspective on street performance

A substantial body of research has inquired into street performance^1–3,7,11,14–20^. This research inspires two psychological questions, which are the focus of this article. The first is concerned with the effect of street performance on the perception or experience of public space. The second is concerned with the audience experience or appreciation of street performance.

#### Effect of street performance on perception of public space

The current literature suggests that the presence of street performance in a public space may enhance the perception or experience of the space. Street performance is a “socially organized” activity^1^. The interactive process made up of a busker’s performing and an audience’s spectating forms a performance place in a public space^3^ or “draws a circle in a square”^7^. Albeit intangible and ephemeral, this performance place creates an atmosphere, which may in turn affect how the surrounding public space is perceived or experienced^15,19^.

Street performance may influence the perception of public space in several ways. First, it may evoke a sense of security or stability. Tanenbaum’s^17^ study on the subways of New York found that street music could facilitate moments of spontaneous contact among strangers or a “transitory community” (p. 105), whereby train riders could feel safer around the stations. Similarly, Doughty and Lagerqvist’s^19^ study on a public square in Stockholm found that street music could “soothe, animate, and soften” the space (p. 59), and could thereby induce senses of inclusion and egalitarianism. Second, street performance may evoke a sense of exploration or change. Harrison-Pepper’s^7^ study on New York’s Washington Square Park found that street performance, as a “dynamic, shifting, breathing event” (p. 127), could stimulate the spatiotemporal configuration of a space. Similarly, Simpson’s^3^ study in Bath found that street performance could interfere with the mundane order of a public space and could thereby make a space more sociable and more convivial. Third, street music in particular may modulate the soundscape of a public space. Street music provides a public space with a soundtrack, on top of the background noise or ambience reflecting the everyday life that is taking place in the space^21^. Moreover, due to its sonic nature, street music can be invisible while omnipresent, and can thereby permeate every corner of a public space without being noticed^18^.

The effect of street performance on the perception of public space has been examined quantitatively. Doubleday^22^ surveyed the visitors to a shopping promenade in Santa Monica. The majority of the respondents perceived street performance as an important feature to the promenade’s attraction and an enhancement to their overall experience of the area. Ho and Au^23^ conducted a field experiment in a public space in Hong Kong to compare pedestrians’ perception of the space without vs. with street music and found that the presence of street music could significantly increase the perceived restorativeness of the space. However, surprisingly, the effect seemed to be more convincing when comparing unengaged passersby vs. engaged audience in the same space with street music. In comparison with unengaged passersby, engaged audience not only perceived the space as significantly more restorative, but they also perceived the space as significantly more visitable and more likeable. One possible interpretation is that the effect of street performance might depend on whether people had truly paid attention to or engaged themselves in a performance. In any case, these studies^22,23^ provide preliminary support for the effect of street performance from a quantitative research perspective.

#### Street audience experience (SAE)

Meanwhile, the current literature also suggests that audience experience or appreciation of street performance can be a complex phenomenon. According to Ho and Au^13^, such a phenomenon can be organized under a *Street Audience Experience (SAE)* framework, which proposes six factors or components regarded as *emotion*, *intellect*, *novelty*, *place*, *interaction*, and *technique*. Emotion captures the emotional or affective responses to a performance (e.g., “*This is a moving performance that really touched my heart.*”). Intellect captures the intellectual or cognitive responses to a performance (e.g., “*This performance conveyed certain message(s) to me.*”). Novelty captures the novelty or originality of a performance in comparison with others (e.g., “*This performance had a newer style compared with those of similar type.*”). Place captures the placemaking quality of a performance (e.g., “*This performance made me “love” this place.*”). Interaction captures a performer’s ability to interact or lead the audience to interact (e.g., “*The performer(s) was good at leading the audience’s emotions.*”). Technique captures a performer’s ability to conduct the performance professionally (e.g., “*The performer(s) had outstanding performance skills.*”).

Ho and Au^13^ have developed and validated an *SAE Scale* through exploratory and confirmatory factor analyses with samples derived from a variety of musical and nonmusical busking. The scale consists of six subscales for measuring the six factors of SAE. The scale can predict outcomes of SAE such as people’s overall satisfaction with a performance and donation to a performance^13,24,25^. Further studies have shown that SAE might be influenced by the audience’s expertise^25^ and familiarity^26^, and so variables as such should be considered when examining SAE.

## Research gaps

Two research gaps have been identified in the reviewed literature. First, there is a shortage of experimental support for the effect of street performance on perception of public space. Most of the quantitative studies did not address the effect experimentally^13,16,22,24–28^, except Ho and Au^23^. For example, Doubleday^22^ was only a basic field survey without systematically comparing absence vs. presence of street performance in a public space or comparing passersby vs. audience in terms of perceiving public space or experiencing street performance. Second, although it is sensible to assume that SAE should account for the effect of street performance on perception of public space, currently there is a lack of empirical data for verifying this assumption. Although Ho and Au^23^ revealed the potential importance of audience engagement, they did not systematically measure or include this variable in their method or analysis. In other words, the mediational role of SAE in the effect of street performance still requires empirical demonstration. Building upon these previous studies^22,23^, the present study takes a field-experimental approach to verify the effect of street performance, while properly assessing the mediating effect of SAE. Integrating the SAE framework into the effect of street performance on perception of public space will address the question of whether such an effect depends on audience engagement with street performance.

### Variables of perception of public space

The key dependent variables (DVs) of the present study are variables related to perceiving or experiencing public space, including *place essentialism*, *place anti-essentialism*, *sonic restorativeness*, and *overall liking*.

#### Place essentialism and anti-essentialism

This study draws on the notion of place essentialism that emerges from environmental perception or environmental aesthetics^29,30^. Place essentialism affords a framework for understanding how a physical setting is turned into a positively appreciated meaningful place. Under this framework, two types of place are differentiated: essentialist and anti-essentialist. An essentialist place is perceived as having an essence or guarded by a spirit; hence, it is experienced as a place of being, stability, history, and tradition. An anti-essentialist place is perceived as constantly evolving or changing; hence, it is experienced as a place of becoming, dynamic, modernity, and progress. Essentialist places promote a sense of protection or security; anti-essentialist places promote opportunities for explorations or changes. Essentialism and anti-essentialism may just be two sets of qualitatively different features that can both be present in a space. The literature review has shown that the presence of street performance could yield senses of stability^17,19^ and dynamic^3,7^ at the same time. Indeed, as Smith^31^ describes, while modern buskers symbolize an ancient tradition (essentialism), the tradition itself is performing outside conventional power structures (anti-essentialism). Hence, it is sensible to expect that street performance could evoke both the essentialist and anti-essentialist qualities of a public space.

#### Sonic restorativeness

Perceived restorativeness refers to the extent to which a setting facilitates recovery from mental fatigue or reflection upon daily stressors^32,33^. A restorative setting holds appealing properties that can draw or direct effortless attention and allow a sense of temporary shift away from present problems. A restorative setting is also coherent in itself and is compatible with the preferences of the people who are experiencing it. The previous field experiment found support for the effect of street performance on restorativeness^23^. Also, it is possible to experience restorativeness from the soundscape of a setting^34^. Hence, it is sensible to expect that street performance could enhance the perceived restorativeness of the soundscape of a public space. For brevity, throughout the remaining sections of this paper, we refer this restorativeness of soundscape to as sonic restorativeness.

#### Overall liking

Overall liking refers to the level of liking or enjoying a particular setting. Previous studies found support for the effect of street performance on this variable^16,22,23^. Hence, it is sensible to expect that street performance could enhance the overall liking of a public space.

### The present study

**Figure 1:**
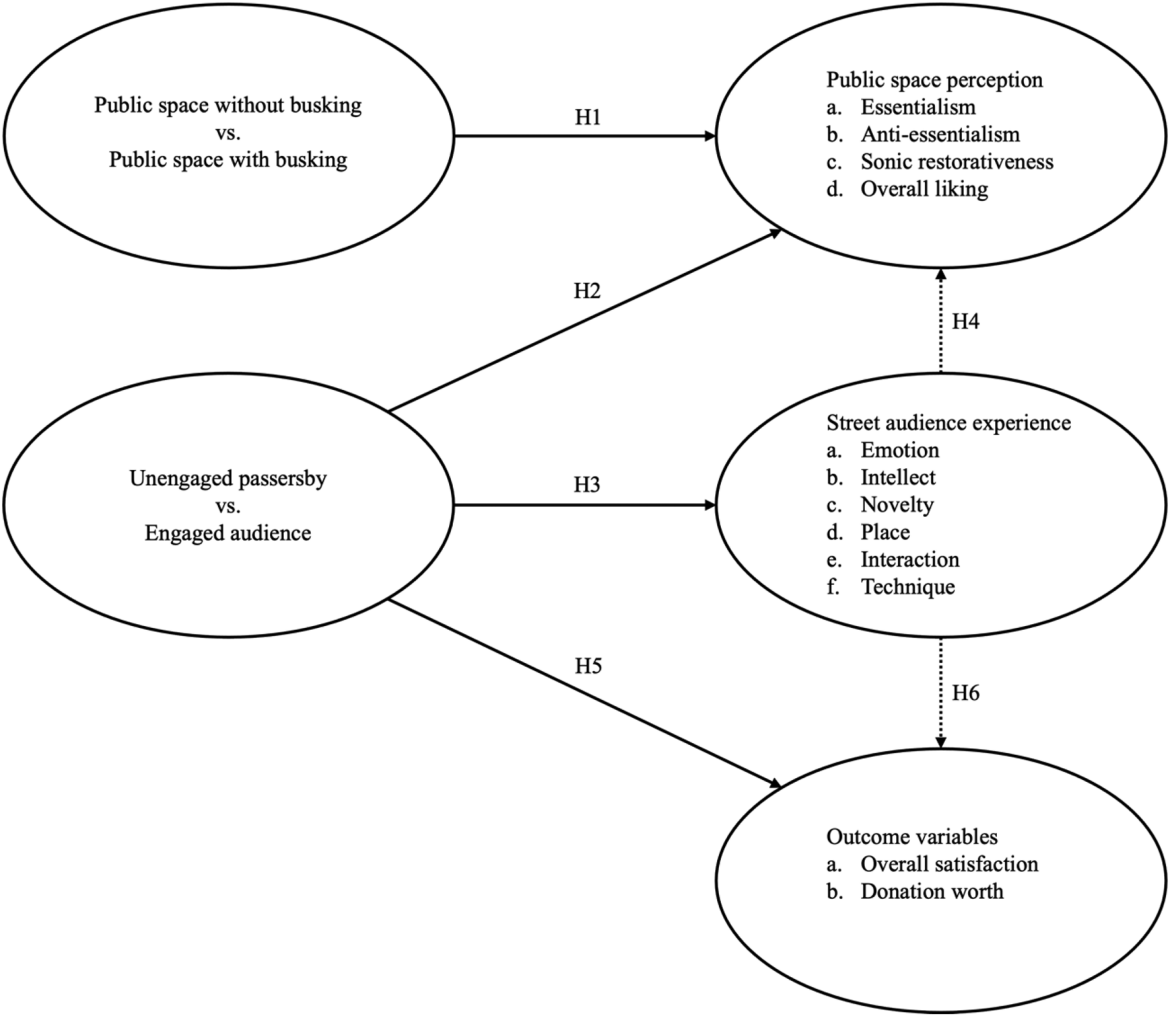
Conceptual graphical representation of research hypotheses. Solid lines denote direct effects; dashed lines denote indirect effects. Image created by the authors.

With a field experiment, the present study aims at verifying the effect of street performance on perception of public space, while examining SAE as a mediator of the effect of street performance. Figure 1 provides a conceptual graphical representation of the research hypotheses of the present study.

To verify the effect of street performance on perception of public space, the following hypotheses are made:

*Public space with busking is perceived as more essentialist (H1a), more anti-essentialist (H1b), more sonically restorative (H1c), and more likeable (H1d) than public space without busking.*

*Engaged audience perceive a public space as more essentialist (H2a), more anti-essentialist (H2b), more sonically restorative (H2c), and more likeable (H2d) than unengaged passersby do.*

To examine the mediating effect of SAE in the effect of street performance, the following hypotheses are made:

*Engaged audience experience more emotion (H3a), more intellect (H3b), more novelty (H3c), more place (H3d), more interaction (H3e), and more technique (H3f) than unengaged passersby do.*

*Engaged audience perceive a public space as more essentialist (H4a), more anti-essentialist (H4b), more sonically restorative (H4c), and more likeable (H4d) than unengaged passersby do through experiencing more positive SAE.*

Finally, the present study considers outcome variables of *overall satisfaction* and *donation worth*. Overall satisfaction refers to people’s satisfaction with a given street performance. Donation worth refers to the amount of money people think is worth tipping or donating to a given street performance. While these variables may seem unrelated to perception of public space, they are effectively related to SAE and street performance in general. Both variables may reflect the psychological consequences of SAE, as previous studies have found positive correlations between these variables and SAE^13,24,25,28^. Furthermore, findings concerning performance satisfaction and donation behavior can be informative to practitioners of street performance or street art in the real world. Hence, the following hypotheses are made:

*Engaged audience are more likely to feel satisfied with a performance (H5a) and perceive the performance as worth donating to (H5b) than unengaged passersby are.*

*Engaged audience are more likely to feel satisfied with a performance (H6a) and perceive the performance as worth donating to (H6b) than unengaged passersby are through experiencing more positive SAE.*

## Method

### Design

A field experiment with a between-subjects design was carried out. This study was a quasi-experiment as random assignment was unfeasible in the field. The key independent variable (IV) was *participant group*, and there were two ways of leveling for this IV. The first compared two levels: *people who were exposed to a public space without busking (control group/condition)* vs. *people who were exposed to a public space with busking (experimental group/condition)*. The second also compared two levels: within a public space with busking, *people who had not stopped to watch the performance (unengaged passersby)* vs. *people who had stopped to watch the performance (engaged audience)*.

**Figure 2:**
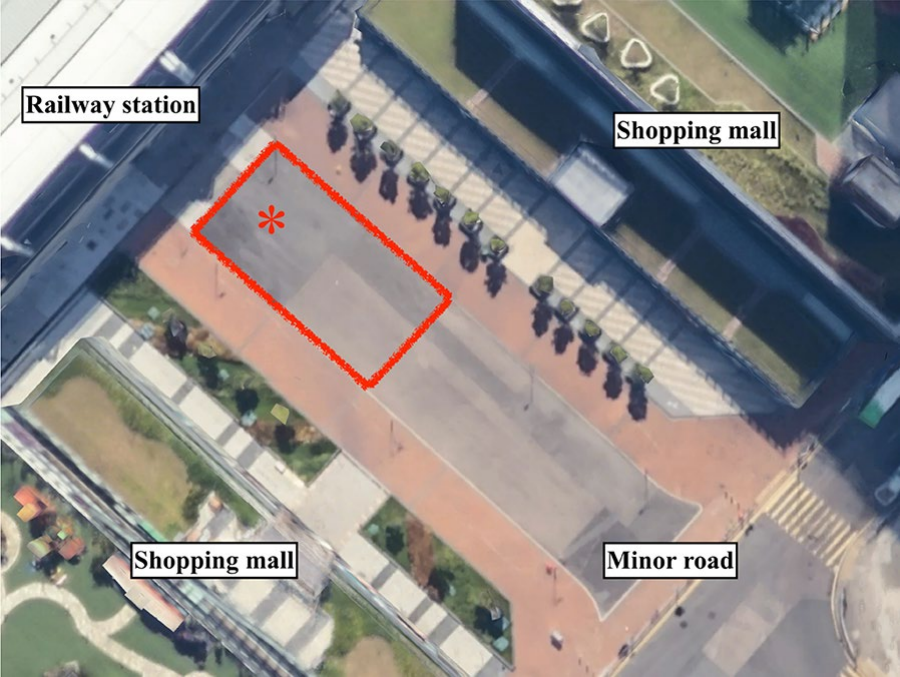
Site of the public space (denoted by a red rectangle) and spot of the street performer (denoted by a red asterisk) for the field experiment. Image generated with Google Maps.

**Figure 3:**
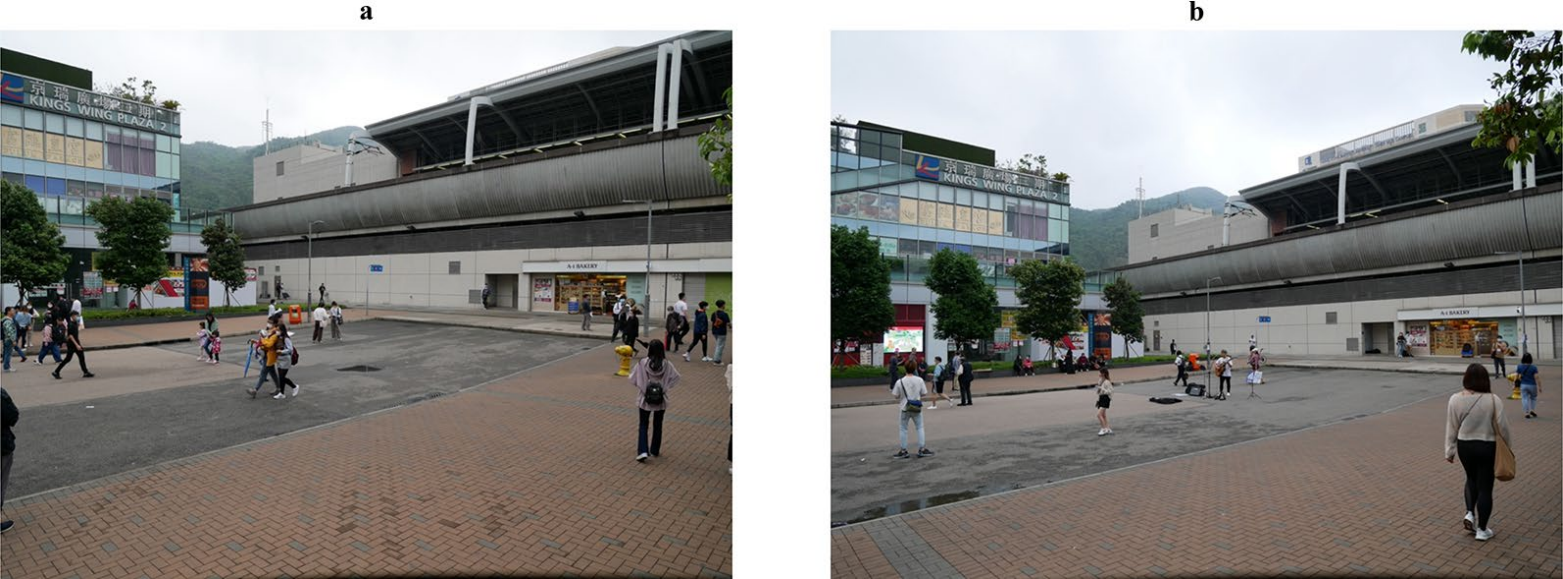
Photographs of the public space without busking (panel **a**) and with busking (panel **b**) during the field experiment. Images created by the authors.

The present study took place in Hong Kong. A public space in a local area known as *Shek Mun* was selected for conducting the field experiment. As shown in Fig. 2, the selected space was an intersection among local shopping malls (from two sides), a railway station (from one side), and a minor road (from one side). In terms of environmental features, the space was 10 m × 20 m in size and rectangular in shape, it had concrete surfaces suitable for pedestrian foot traffic, and its surfaces were generally flat without slopes or platforms. In terms of acoustic features, the space was open without any covers on the top, its ambience was composed of its immediate pedestrians, shops, and road traffics, and there were no external sound systems from the surrounding buildings. This selected space was frequented by foot traffic of local commuters. As illustrated in Fig. 3, there were no more than 10 people inside the space at any given moment during the study period, and there were no pedestrian congestions throughout the study. While other widespread public spaces such as squares and parks could have been adopted in theory, they were unfeasible in practice. Although there is a major square in Hong Kong (i.e., Times Square), street performance in that square has been banned since 2018^35,36^. Street performance is generally prohibited in parks in Hong Kong. To legitimately conduct street performance in Hong Kong, a permit must be obtained from an authority prior to the event. The public space adopted for the present study was actually permitted by the Hong Kong Police Force (www.police.gov.hk). All things considered, this selected space should appropriately represent a local public space suitable for street performance.

### Participants

In total, 95 (31.4%), 98 (32.3%), and 110 (36.3%) responses were collected on April 2 (control condition), 16, and 23 (experimental condition), 2023, respectively. After discarding four, five, and two (April 2, 16, and 23) invalid responses, 292 (96.4%) valid responses were retained. A majority of 253 participants (86.6%) had resided locally for at least seven years, representing a local population. Table 1 summarizes the participants’ demographics. In all, there were 91 (31.2%) control and 201 (68.8%) experimental participants. Among the experimental participants, there were 74 (36.8%) unengaged passersby and 127 (63.2%) engaged audience. Among the engaged audience, 10 (7.9%) watched the performance for up to 1 min, 53 (41.7%) up to 5 min, 36 (28.3%) up to 10 min, 14 (11.0%) up to 15 min, six (4.7%) up to 20 min, and eight (6.3%) more than 20 min.

**Table 1:** Participants’ gender distribution and mean years of age.

	Control	Experimental: unengaged passerby	Experimental: engaged audience	Total
Female	54	34	65	153
Years of age (*SD*)	41.1 (14.0)	34.9 (13.7)	34.5 (11.3)	36.9 (13.1)
Male	37	38	61	136
Years of age (*SD*)	37.3 (17.7)	33.4 (13.3)	37.2 (13.6)	36.1 (14.8)
Prefer not to say	0	2	1	3
Years of age (*SD*)	–	–	–	–
Gender collapsed	91	74	127	292
Years of age (*SD*)	39.5 (15.7)	34.1 (13.4)	35.8 (12.5)	36.5 (13.9)

Participants who did not report gender also did not report age.

### Street performance

The majority of street performances in Hong Kong is musical, and Hong Kong street musicians typically perform Hong Kong pop, a music genre familiar to locals^37^. For the experimental condition, a solo Hong Kong male street musician with 10 years of busking experience was hired to perform Hong Kong pop songs in the public space. He sang and played an acoustic guitar through a portable, battery-powered amplifier at a constant volume level. In setting the volume level, we referred to the Melbourne Busking Handbook^38^, given that this Australian city is reputable for best practices of street performance^39^. The maximum level was set to be slightly above the ambience. At the same time, we ensured that this volume was not too loud that people would have to strain their voices to hear themselves above the performance. Furthermore, we ensured that this volume could not drown the sounds of other activities when a person was 30 m away from the performance.

For variety of songs, the busker was asked to prepare two sets of songs, one intended to convey a positive/happy mood and the other one negative/sad. The busker proposed eight positive songs and six negative songs. All songs were pretested to ensure that the intended moods were aligned with the perceived moods. Details of the pretest are presented in Supplementary Method and Table S1 online. In the actual performance, both sets lasted approximately 30 min. The two sets of songs were performed alternately throughout the experimental condition. On April 16, a negative–positive–negative–positive order was performed. On April 23, a positive–negative–positive–negative order was performed. Thus, a total of 4 × 2 = 8 sets were performed in the experimental condition. The busker took a 15-min break after each set. No data were collected during the breaks. In total, among the experimental participants, 107 (54.3%) and 90 (45.7%) participated during the positive- and negative-mood sessions, respectively (four missing data). However, mood of songs is *not* a focus of this study. This variable was only included in the interest of ecological validity, as buskers perform songs of various emotions in the real world. Also, varying mood of songs allowed us to rule out the effect of this variable.

### Measures

Measures are summarized in Table 2. Place essentialism and place anti-essentialism were measured with 10 scale items (five items each) obtained from Lewicka et al.^30^. Sonic restorativeness was measured with a nine-item Perceived Restorativeness Soundscape Scale proposed by Payne^34^. Overall liking was measured with a four-item scale used in previous studies^16,23^. The six factors of SAE were measured with a 24-item (four items each) SAE Scale proposed by Ho and Au^13^. For these measures, composite scores were computed by simple unit weighting. As summarized in Table 2, all measures achieved Cronbach’s αs > 0.80. Overall satisfaction and donation worth were measured with single items. As control measures, the participants’ *frequency of visit*, *expertise*, *familiarity*, and *interest* were also measured, with single items. Specifically, according to Leder et al.^40^, expertise, familiarity, and interest can influence aesthetic appreciation and aesthetic judgments. Hence, since SAE is concerned, these variables were controlled for. All items were measured on a 7-point scale.

**Table 2:** Scale items.

**Place essentialism**^**1, 5**^ **(Cronbach’s α = .815)**
One can easily feel a part of this place, this place draws in with its atmosphere.
One can feel history in this place.
This place has a soul, this is a climatic place.
This place has a character, this place is unique.
This place offers rest, you can stop in this place.
**Place anti-essentialism**^**1, 5**^ **(Cronbach’s α = .816)**
This is a modern place.
This is a dynamic place, its character changes.
This place encourages activity, there are many things going on in this place.
This place is open to strangers.
Different life styles and ways of life unite in this place.
**Sonic restorativeness**^**2, 5**^ **(Cronbach’s α = .947)**
When I hear the sounds of this place I feel free from work, routine and responsibilities.
The sonic environment here is a refuge from unwanted distractions.
The sounds I am hearing here seem to fit together quite naturally with this place.
The sonic environment here fits with my personal preferences.
The sounds of this place make me want to linger here.
The sounds of this place make me wonder about things.
I find the sonic environment here appealing.
I am engrossed by the sonic environment here.
The sonic environment here suggests the size of this place is limitless.
**Overall liking**^**3, 5**^ **(Cronbach’s α = .959)**
I like this place a great deal.
I like this place very much.
I would enjoy this place a lot.
I would really enjoy this place.
**SAE emotion**^**4, 5**^ **(Cronbach’s α = .931)**
This is a moving performance that really touched my heart.
This performance alleviated my stress and made me feel relaxed.
This performance helped me release my emotions.
This performance made me feel engaged as if I had entered another world.
**SAE intellect**^**4, 5**^ **(Cronbach’s α = .893)**
This performance conveyed certain message(s) to me.
This was an inspiring performance; I came up with new idea about something.
This was a stimulating performance that made me reflect on something.
This performance made me think of something (e.g., events or people).
**SAE novelty**^**4, 5**^ **(Cronbach’s α = .919)**
This performance had a newer style compared with those of similar type.
This performance was more creative than those of similar type.
This performance was more unique than those of similar type.
This performance made me feel fresh compared with those of similar type.
**SAE place**^**4, 5**^ **(Cronbach’s α = .914)**
This performance made me “love” this place.
This performance made me feel I belonged to this place.
This performance made me feel my connection with this place.
This performance made this place feel secure.
**SAE interaction**^**4, 5**^ **(Cronbach’s α = .889)**
The performer(s) was good at leading the audience’s emotions.
The performer(s) consciously used eye contact to enrich the performance.
The performer(s) interacted with the audience/made the audience interact.
The performer(s) was paying attention to how the audience reacted.
**SAE technique**^**4, 5**^ **(Cronbach’s α = .903)**
The performer(s) had outstanding performance skills.
This was a performance of professional standards.
This performer(s) most likely has undertaken proper performance training.
This performer(s) most likely has spent a great deal of time on practicing.
**Overall satisfaction**^**6**^
Overall speaking, how satisfied are you with this street performance?
**Donation worth**^**7**^
How much money do you think this performance is worth as donation (per donor)?
**Frequency of visit**^**8**^
How frequently do you walk past this place?
**Expertise**^**9**^
How much are you an expert on pop music (e.g., you have studied or produced pop music)?
**Familiarity**^**9**^
How familiar are you with the music genre performed in this street performance?
**Interest**^**9**^
How interested are you in pop music?

^1^ Lewicka et al.^30^.

^2^ Payne^34^.

^3^ Ho and Au^16,23^.

^4^ Ho and Au^13^.

^5^ From 1 = *Strongly disagree* to 7 = *Strongly agree.*

^6^ From 1 = *Completely unsatisfied* to 7 = *Completely satisfied.*

^7^ 1 = *HK$0*, 2 = *Less than HK$10*, 3 = *HK$10 or more*, 4 = *HK$20 or more*, 5 = *HK$50 or more*, 6 = *HK$100 or more*, 7 = *HK$200 or more.*

^8^ From 1 = *Never* to 7 = *Always.*

^9^ From 1 = *Not at all* to 7 = *Extremely.*

### Procedure

The study was carried out on three separate Sundays, April 2, 16, and 23, 2023. The control condition took place on April 2 and the experimental 16 and 23. On all three days, street surveys were administered to people passing by the public space during 2:30–5:30 p.m. All participants were asked to assess their perception of the public space as well as frequency of visit. In the experimental condition, both unengaged passersby and engaged audience were additionally asked to assess their SAE and outcomes of SAE as well as expertise, familiarity, and interest. Each participant received 30 Hong Kong dollars to appreciate or compensate for their time and effort.

Ethical approval was obtained from the Human Research Ethics Committee of The Education University of Hong Kong. Informed consent was obtained from all participants prior to any data collection. The study was conducted in accordance with the Declaration of Helsinki.

### Statistical analyses

Analyses were conducted using IBM SPSS Statistics. As shown in Supplementary Results online, preliminary analyses found significant effects of participant group on frequency of visit, expertise, familiarity, and interest. Hence, in the main analyses, these variables were specified as covariates where public space perception (frequency of visit) and SAE (expertise, familiarity, and interest) were concerned.

To test the effect of street performance on public space perception (H1–2), one-way ANOVAs with participant group as a between-subjects factor were run on essentialism, anti-essentialism, sonic restorativeness, and overall liking, respectively, as the DV. Then, a priori contrasts per ANOVA were examined. For H1, the contrasts compared the control vs. experimental (unengaged passersby and engaged audience combined) groups, by specifying coefficients of –1 for the control group, 0.5 for unengaged passersby, and 0.5 for engaged audience. For H2, the contrasts compared unengaged passersby vs. engaged audience within the experimental group, by specifying coefficients of 0 for the control group, –1 for unengaged passersby, and 1 for engaged audience. To confirm the results while controlling for frequency of visit, ANCOVAs specifying this variable as a covariate were run on all DVs again. For H1, the analysis was one-way by specifying control vs. experimental groups as a between-subjects factor. For H2, the analysis was two-way by specifying unengaged passersby vs. engaged audience and mood of songs as between-subjects factors.

To test the effect of street performance on SAE (H3) and outcome variables (H5), two-way ANCOVAs with unengaged passersby vs. engaged audience and mood of songs as between-subjects factors and expertise, familiarity, and interest as covariates were run on SAE emotion, intellect, novelty, place, interaction, technique (H3), overall satisfaction, and donation worth (H5), respectively, as the DV.

To test the mediating effect of SAE in the effect of street performance on public space perception (H4) and outcome variables (H6), mediation analyses with PROCESS (model 4)^41^ specifying unengaged passersby vs. engaged audience as the IV, SAE emotion, intellect, novelty, place, interaction, and technique as parallel mediators, and frequency of visit (H4 only), expertise, familiarity, and interest as covariates were run on essentialism, anti-essentialism, sonic restorativeness, overall liking (H4), overall satisfaction, and donation worth (H6), respectively, as the DV.

To determine statistical significance for ANOVAs or ANCOVAs, we generally adopted a *p* value of 0.05. However, where multiple tests were conducted to test a given hypothesis, this *p* value was adjusted according to the Bonferroni method, by dividing this *p* value by the number of multiple tests, depending on the total number of DVs and comparisons. To determine statistical significance for mediation analyses, we adopted a 95% confidence interval (CI). If the CI included zero, the result would be determined as nonsignificant. If the CI excluded zero, the result would be determined as significant.

## Results

### Effect of street performance on perception of public space

Based on an adjusted *p* value of 0.05 ÷ 4 DVs = 0.0125, one-way ANOVAs were significant in essentialism (*F*(2, 289) = 10.41, *p* < 0.001, partial η^2^ = 0.07), anti-essentialism (*F*(2, 289) = 7.85, *p* < 0.001, partial η^2^ = 0.05), sonic restorativeness (*F*(2, 287) = 23.07, *p* < 0.001, partial η^2^ = 0.14), and overall liking (*F*(2, 287) = 8.08, *p* < 0.001, partial η^2^ = 0.05).

#### Control vs. experimental groups (H1)

**Figure 4:**
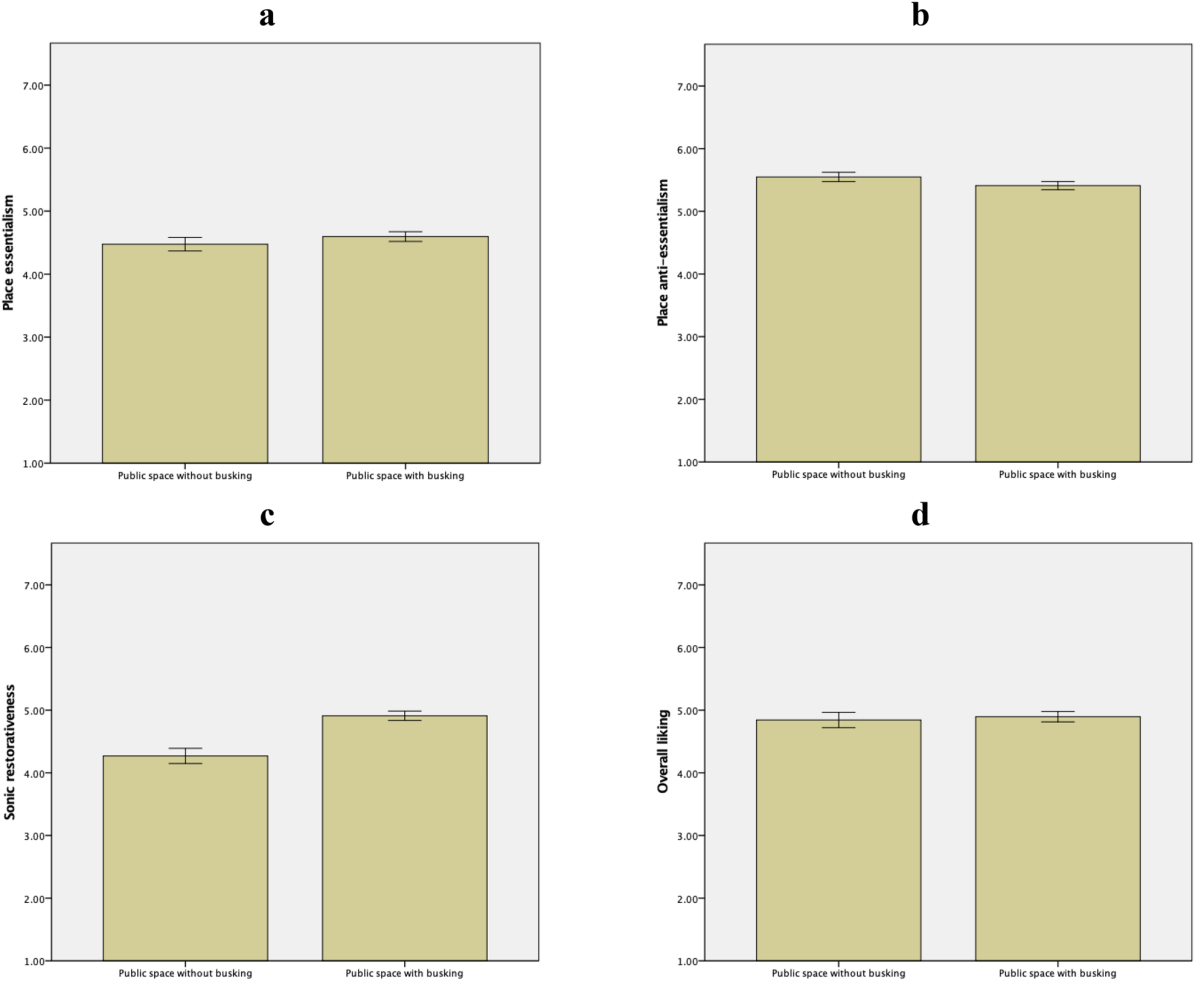
Descriptive statistics of perception of public space presented as public space without busking vs. public space with busking with error bars of ± 1 *SE*. Essentialism (panel **a**): *M*_without_ = 4.48, *SD* = 1.03; *M*_with_ = 4.60, *SD* = 1.09. Anti-essentialism (panel **b**): *M*_without_ = 5.55, *SD* = 0.71; *M*_with_ = 5.41, *SD* = 0.92. Sonic restorativeness (panel **c**): *M*_without_ = 4.27, *SD* = 1.16; *M*_with_ = 4.91, *SD* = 1.05. Overall liking (panel **d**): *M*_without_ = 4.84, *SD* = 1.16; *M*_with_ = 4.90, *SD* = 1.16. Graphs generated with IBM SPSS Statistics.

Descriptive statistics and a priori contrasts are summarized in Fig. 4 and Table 3 (upper panel), respectively. The control vs. experimental groups did not differ significantly in essentialism, anti-essentialism, nor overall liking. However, the two groups differed significantly in sonic restorativeness. In sum, public space with busking was perceived as more sonically restorative than public space without busking. Thus, while H1a, H1b, and H1d were not supported, H1c was supported. These results remained unchanged after controlling for frequency of visit, as supported by the ANCOVAs summarized in Table 4 (upper panel).

**Table 3:** A priori contrasts on perception of public space.

Public space without busking vs. public space with busking				
DV	*t*	*df*	*p*	*d*
Essentialism	0.23	289	.815	.11
Anti-essentialism	− 1.85	289	.065	.17
**Sonic restorativeness**	**4.02**	**287**	**.000**	**.58**
Overall liking	− 0.28	287	.783	.05

Unengaged passersby vs. engaged audience				
DV	*t*	*df*	*p*	*d*
**Essentialism**	**4.47**	**289**	**.000**	**.63**
**Anti-essentialism**	**3.74**	**289**	**.000**	**.52**
**Sonic restorativeness**	**4.76**	**287**	**.000**	**.74**
**Overall liking**	**4.00**	**287**	**.000**	**.59**

*p* value for statistical significance is adjusted to .05 ÷ (4 DVs × 2 contrasts) = .00625; significant result is bold.

**Table 4:** ANCOVAs on perception of public space.

Public space without busking vs. public space with busking					
Effect	DV	*F*	*df*	*p*	Partial η^2^
Frequency of visit	Essentialism	4.80	1, 286	.029	.02
	**Anti-essentialism**	**11.92**	**1, 286**	**.001**	**.04**
	Sonic restorativeness	2.89	1, 286	.090	.01
	**Overall liking**	**13.10**	**1, 286**	**.000**	**.04**
Without busking vs. with busking	Essentialism	1.89	1, 286	.171	.01
	Anti-essentialism	0.30	1, 286	.587	.00
	**Sonic restorativeness**	**24.45**	**1, 286**	**.000**	**.08**
	Overall liking	1.23	1, 286	.269	.00

Unengaged passersby vs. engaged audience					
Effect	DV	*F*	*df*	*p*	Partial η^2^
Frequency of visit	Essentialism	0.13	1, 189	.717	.00
	Anti-essentialism	2.81	1, 189	.095	.02
	Sonic restorativeness	0.16	1, 189	.690	.00
	Overall liking	3.63	1, 189	.058	.02
Passersby vs. audience	**Essentialism**	**15.95**	**1, 189**	**.000**	**.08**
	**Anti-essentialism**	**9.97**	**1, 189**	**.002**	**.05**
	**Sonic restorativeness**	**23.78**	**1, 189**	**.000**	**.11**
	**Overall liking**	**13.46**	**1, 189**	**.000**	**.07**
Mood of songs	Essentialism	0.03	1, 189	.865	.00
	Anti-essentialism	3.20	1, 189	.075	.02
	Sonic restorativeness	0.35	1, 189	.555	.00
	Overall liking	0.23	1, 189	.629	.00
Passersby vs. audience × mood of songs	Essentialism	0.54	1, 189	.465	.00
	Anti-essentialism	0.36	1, 189	.552	.00
	Sonic restorativeness	0.01	1, 189	.935	.00
	Overall liking	0.98	1, 189	.324	.01

*p* value for statistical significance is adjusted to .05 ÷ (4 DVs × 2 contrasts) = .00625; significant result is bold.

#### Unengaged passersby vs. engaged audience (H2)

**Figure 5:**
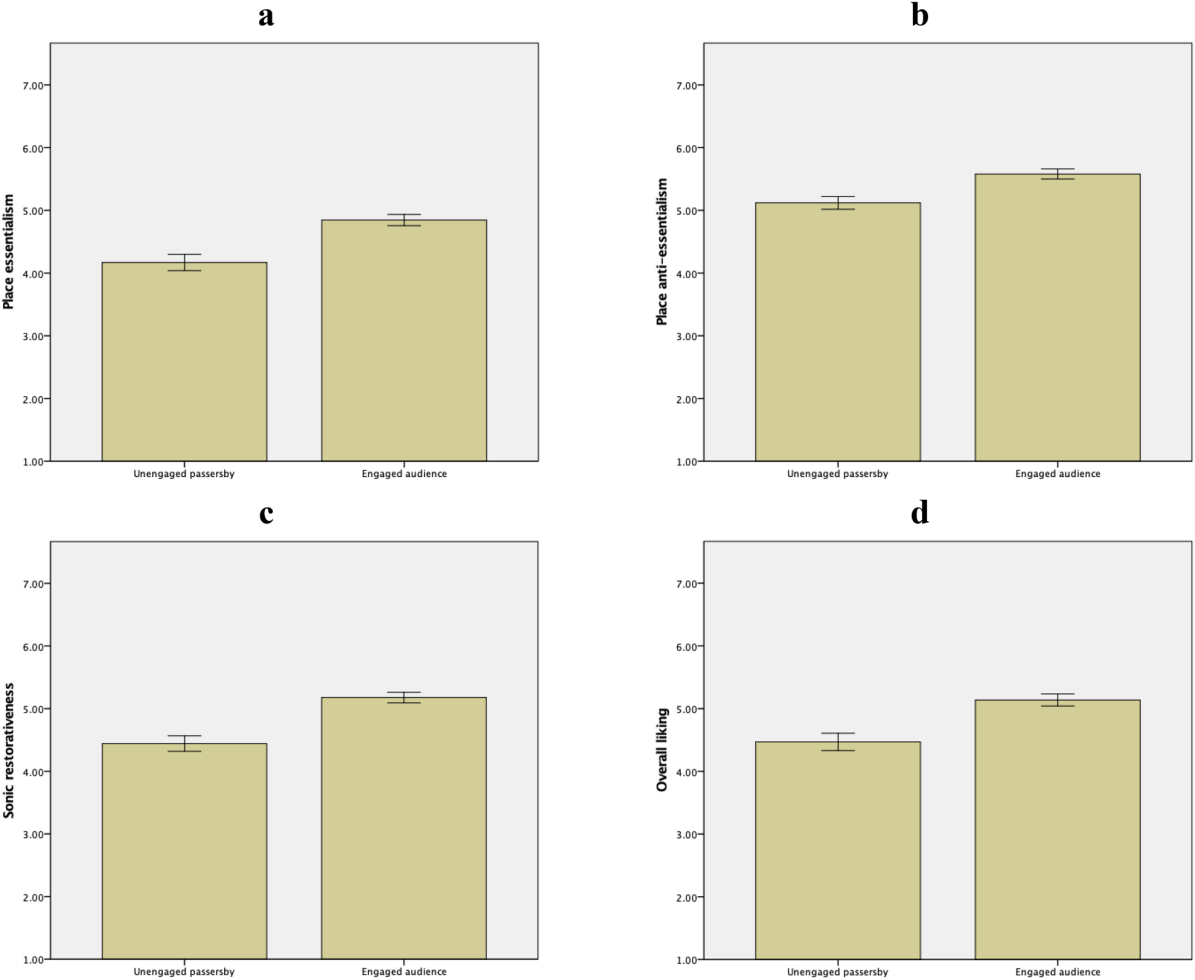
Descriptive statistics of perception of public space presented as unengaged passersby vs. engaged audience with error bars of ± 1 *SE*. Essentialism (panel **a**): *M*_passersby_ = 4.17, *SD* = 1.11; *M*_audience_ = 4.84, *SD* = 1.00. Anti-essentialism (panel **b**): *M*_passersby_ = 5.12, *SD* = 0.87; *M*_audience_ = 5.58, *SD* = 0.91. Sonic restorativeness (panel **c**): *M*_passersby_ = 4.44, *SD* = 1.05; *M*_audience_ = 5.18, *SD* = 0.96. Overall liking (panel **d**): *M*_passersby_ = 4.47, *SD* = 1.17; *M*_audience_ = 5.14, *SD* = 1.09. Graphs generated with IBM SPSS Statistics.

Descriptive statistics and a priori contrasts are summarized in Fig. 5 and Table 3 (lower panel), respectively. Unengaged passersby vs. engaged audience differed significantly in essentialism, anti-essentialism, sonic restorativeness, and overall liking. In sum, engaged audience perceived the public space as more essentialist, more anti-essentialist, more sonically restorative, and more likeable than unengaged passersby did. Thus, H2a, H2b, H2c, and H2d were all supported. These results remained unchanged after controlling for frequency of visit and were unaffected by mood of songs, as supported by the ANCOVAs summarized in Table 4 (lower panel).

### SAE as a mediator in the effect of street performance on perception of public space

#### Effect of street performance on SAE (H3)

**Figure 6:**
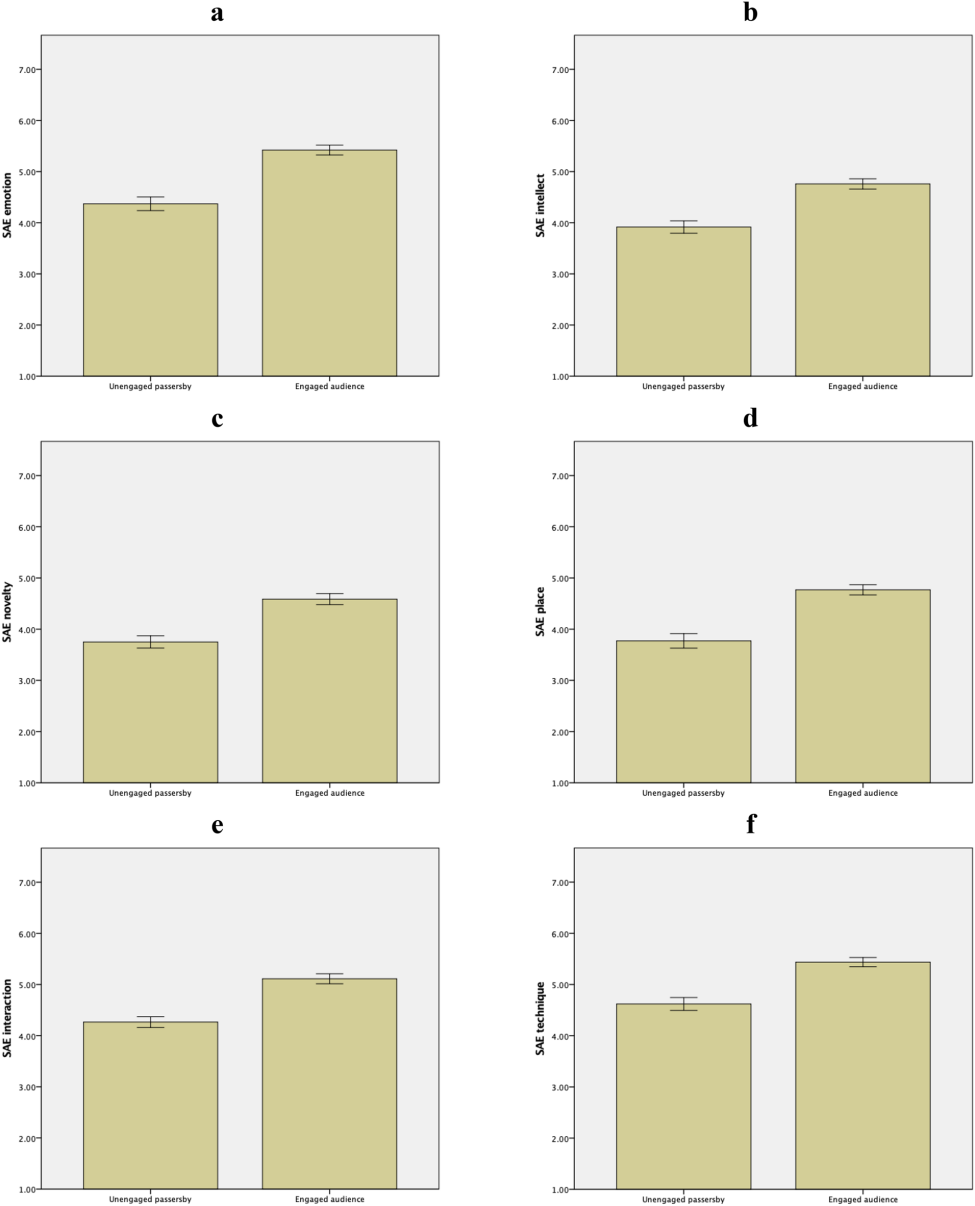
Descriptive statistics of SAE presented as unengaged passersby vs. engaged audience with error bars of ± 1 *SE*. Emotion (panel **a**): *M*_passersby_ = 4.37, *SD* = 1.15; *M*_audience_ = 5.42, *SD* = 1.09. Intellect (panel **b**): *M*_passersby_ = 3.92, *SD* = 1.05; *M*_audience_ = 4.76, *SD* = 1.13. Novelty (panel **c**): *M*_passersby_ = 3.75, *SD* = 1.03; *M*_audience_ = 4.59, *SD* = 1.21. Place (panel **d**): *M*_passersby_ = 3.77, *SD* = 1.22; *M*_audience_ = 4.77, *SD* = 1.12. Interaction (panel **e**): *M*_passersby_ = 4.26, *SD* = 0.91; *M*_audience_ = 5.11, *SD* = 1.10. Technique (panel **f**): *M*_passersby_ = 4.62, *SD* = 1.08; *M*_audience_ = 5.44, *SD* = 1.02. Graphs generated with IBM SPSS Statistics.

Descriptive statistics and ANCOVAs are summarized in Fig. 6 and Table 5, respectively. The differences between unengaged passersby vs. engaged audience were significant in all six factors of SAE. In sum, engaged audience experienced more emotion, more intellect, more novelty, more place, more interaction, and more technique than unengaged passersby did. Mood of songs had no significant effects. Thus, H3a, H3b, H3c, H3d, H3e, and H3f. were all supported.

**Table 5:** ANCOVAs of unengaged passersby vs. engaged audience and mood of songs on SAE.

Effect	DV	*F*	*df*	*p*	Partial η^2^
Expertise	Emotion	0.17	1, 190	.679	.00
	Intellect	0.71	1, 190	.402	.00
	Novelty	1.14	1, 190	.286	.01
	Place	1.39	1, 190	.240	.01
	Interaction	0.00	1, 190	.980	.00
	Technique	0.24	1, 190	.627	.00
Familiarity	Emotion	0.44	1, 190	.506	.00
	Intellect	0.72	1, 190	.397	.00
	Novelty	0.01	1, 190	.934	.00
	Place	0.04	1, 190	.850	.00
	Interaction	0.07	1, 190	.797	.00
	Technique	1.09	1, 190	.297	.01
Interest	Emotion	0.31	1, 190	.576	.00
	Intellect	0.67	1, 190	.414	.00
	Novelty	0.01	1, 190	.940	.00
	Place	5.11	1, 190	.025	.03
	Interaction	4.60	1, 190	.033	.02
	**Technique**	**9.56**	**1, 190**	**.002**	**.05**
Passersby vs. audience	**Emotion**	**35.03**	**1, 190**	**.000**	**.16**
	**Intellect**	**20.55**	**1, 190**	**.000**	**.10**
	**Novelty**	**20.14**	**1, 190**	**.000**	**.10**
	**Place**	**25.63**	**1, 190**	**.000**	**.12**
	**Interaction**	**24.49**	**1, 190**	**.000**	**.11**
	**Technique**	**20.05**	**1, 190**	**.000**	**.10**
Mood of songs	Emotion	1.09	1, 190	.297	.01
	Intellect	1.75	1, 190	.188	.01
	Novelty	0.05	1, 190	.833	.00
	Place	1.00	1, 190	.318	.01
	Interaction	0.01	1, 190	.915	.00
	Technique	0.01	1, 190	.929	.00
Passersby vs. audience × mood of songs	Emotion	0.79	1, 190	.374	.00
	Intellect	0.29	1, 190	.591	.00
	Novelty	0.01	1, 190	.923	.00
	Place	0.00	1, 190	.961	.00
	Interaction	0.41	1, 190	.522	.00
	Technique	0.32	1, 190	.575	.00

*p* value for statistical significance is adjusted to .05 ÷ 6 DVs = .0083; significant result is bold.

A significant effect of interest on technique was noted. People with higher interest in music might be more sensitive to or aware of the agent or proxy producing or delivering the music, i.e., the street performer. Hence, people with higher interest might be more likely to pay attention to and appreciate the performer’s technique.

#### Effect of street performance on perception of public space through SAE (H4)

Mediation analyses are summarized in Table 6.

**Table 6:** Mediation analyses of unengaged passersby vs. engaged audience on perception of public space through SAE.

DV = Essentialism				
Effect	*b*	*SE*	95% LLCI	95% ULCI
Direct effect	0.06	0.14	− 0.22	0.34
Indirect effect				
**Total**	**0.48**	**0.11**	**0.27**	**0.71**
Emotion	− 0.06	0.10	− 0.26	0.14
Intellect	0.02	0.07	− 0.12	0.17
**Novelty**	**0.16**	**0.07**	**0.04**	**0.32**
**Place**	**0.31**	**0.10**	**0.14**	**0.52**
Interaction	− 0.01	0.07	− 0.16	0.12
Technique	0.06	0.06	− 0.06	0.18

DV = Anti-essentialism				
Effect	*b*	*SE*	95% LLCI	95% ULCI
Direct effect	0.04	0.14	− 0.23	0.31
Indirect effect				
**Total**	**0.29**	**0.09**	**0.13**	**0.48**
Emotion	0.00	0.09	− 0.17	0.19
Intellect	0.06	0.06	− 0.06	0.18
Novelty	− 0.01	0.06	− 0.12	0.11
Place	0.11	0.07	− 0.01	0.25
Interaction	0.01	0.07	− 0.13	0.14
Technique	0.12	0.07	0.00	0.26

DV = Sonic restorativeness				
Effect	*b*	*SE*	95% LLCI	95% ULCI
Direct effect	− 0.01	0.12	− 0.24	0.22
Indirect effect				
**Total**	**0.65**	**0.14**	**0.40**	**0.94**
**Emotion**	**0.20**	**0.10**	**0.02**	**0.41**
Intellect	0.05	0.06	− 0.06	0.18
Novelty	− 0.03	0.06	− 0.14	0.08
**Place**	**0.25**	**0.08**	**0.12**	**0.41**
Interaction	0.09	0.06	− 0.03	0.21
Technique	0.09	0.06	− 0.02	0.23

DV = Overall liking				
Effect	*b*	*SE*	95% LLCI	95% ULCI
Direct effect	− 0.12	0.14	− 0.40	0.16
Indirect effect				
**Total**	**0.64**	**0.14**	**0.38**	**0.93**
Emotion	0.20	0.13	− 0.03	0.47
Intellect	− 0.04	0.06	− 0.18	0.07
Novelty	− 0.01	0.06	− 0.12	0.10
**Place**	**0.35**	**0.11**	**0.16**	**0.59**
Interaction	0.12	0.08	− 0.03	0.28
Technique	0.03	0.08	− 0.12	0.19

Significant result is bold.

Regarding essentialism, the direct effect was nonsignificant, while the total indirect effect of SAE was significant. Specifically, individual factors of novelty and place had significant indirect effects.

Regarding anti-essentialism, the direct effect was nonsignificant, while the total indirect effect of SAE was significant. No significant indirect effects of individual factors were found.

Regarding sonic restorativeness, the direct effect was nonsignificant, while the total indirect effect of SAE was significant. Specifically, individual factors of emotion and place had significant indirect effects.

Regarding overall liking, the direct effect was nonsignificant, while the total indirect effect of SAE was significant. Specifically, individual factor of place had a significant indirect effect.

In sum, SAE fully mediated the effect of street performance on perception of public space. Thus, H4a, H4b, H4c, and H4d were all supported.

### SAE as a mediator in the effect of street performance on outcome variables

#### Effect of street performance on outcome variables (H5)

**Figure 7:**
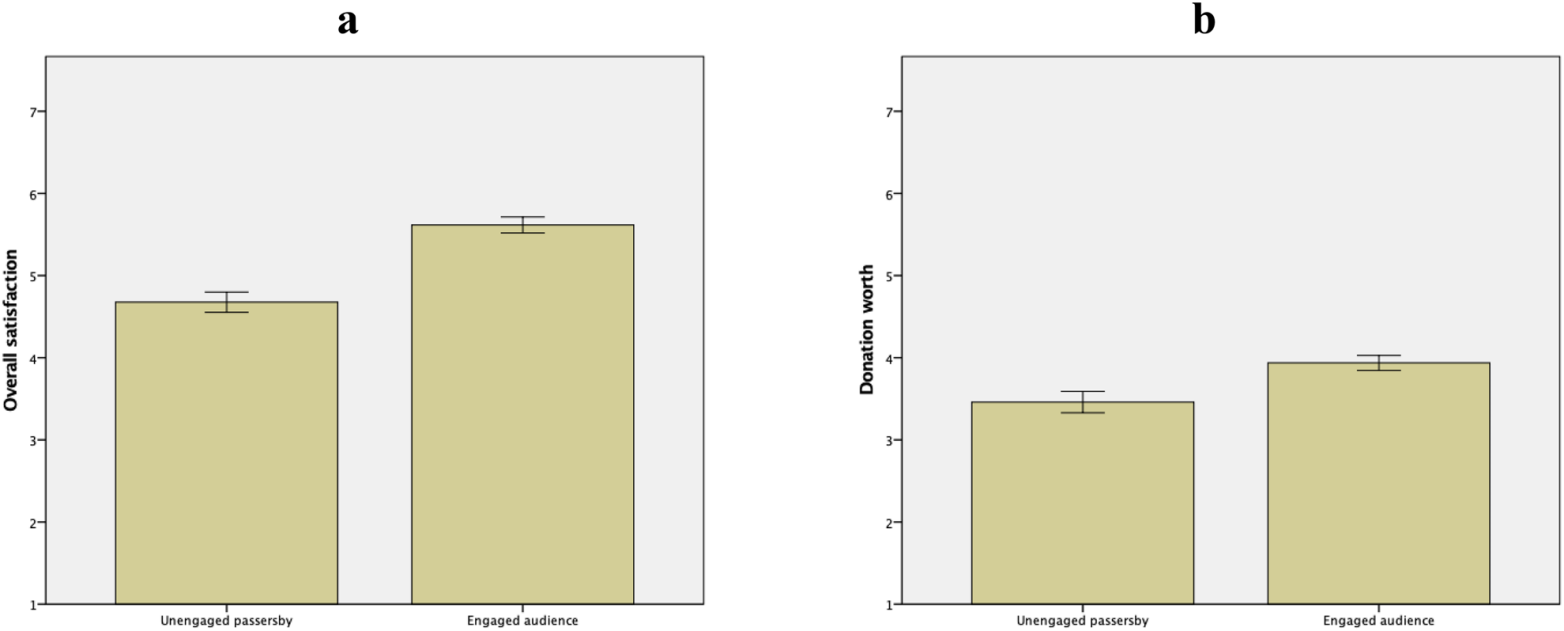
Descriptive statistics of outcome variables presented as unengaged passersby vs. engaged audience with error bars of ± 1 *SE*. Overall satisfaction (panel **a**): *M*_passersby_ = 4.68, *SD* = 1.06; *M*_audience_ = 5.62, *SD* = 1.08. Donation worth (panel **b**): *M*_passersby_ = 3.46, *SD* = 1.12; *M*_audience_ = 3.94, *SD* = 1.02. Graphs generated with IBM SPSS Statistics.

Descriptive statistics and ANCOVAs are summarized in Fig. 7 and Table 7, respectively. The differences between unengaged passersby vs. engaged audience were significant in both overall satisfaction and donation worth. In sum, engaged audience were more likely to feel satisfied with the performance and perceive the performance as worth donating to than unengaged passersby were. Mood of songs had no significant effects. Thus, H5a and H5b were both supported.

**Table 7:** ANCOVAs of unengaged passersby vs. engaged audience and mood of songs on outcome variables.

Effect	DV	*F*	*df*	*p*	Partial η^2^
Expertise	Overall satisfaction	0.25	1, 188	.615	.00
	Donation worth	0.59	1, 188	.443	.00
Familiarity	Overall satisfaction	0.02	1, 188	.879	.00
	Donation worth	0.00	1, 188	.989	.00
Interest	Overall satisfaction	4.44	1, 188	.036	.02
	**Donation worth**	**10.89**	**1, 188**	**.001**	**.06**
Passersby vs. audience	**Overall satisfaction**	**26.52**	**1, 188**	**.000**	**.12**
	**Donation worth**	**7.04**	**1, 188**	**.009**	**.04**
Mood of songs	Overall satisfaction	0.42	1, 188	.520	.00
	Donation worth	4.57	1, 188	.034	.02
Passersby vs. audience × mood of songs	Overall satisfaction	0.00	1, 188	.979	.00
	Donation worth	0.01	1, 188	.935	.00

*p* value for statistical significance is adjusted to .05 ÷ 2 DVs = .025; significant result is bold.

A significant effect of interest on donation worth was noted. As mentioned earlier, people with higher interest might be more sensitive to the agent producing the music. Hence, these people might be more likely to think of the street performer as deserving of donations.

#### Effect of street performance on outcome variables through SAE (H6)

Mediation analyses are summarized in Table 8.

**Table 8:** Mediation analyses of unengaged passersby vs. engaged audience on outcome variables through SAE.

DV = Overall satisfaction				
Effect	*b*	*SE*	95% LLCI	95% ULCI
Direct effect	0.08	0.13	− 0.18	0.34
Indirect effect				
**Total**	**0.75**	**0.13**	**0.51**	**1.01**
**Emotion**	**0.41**	**0.12**	**0.20**	**0.66**
Intellect	− 0.01	0.06	− 0.13	0.11
Novelty	0.04	0.06	− 0.08	0.15
Place	0.08	0.07	− 0.04	0.23
Interaction	0.06	0.07	− 0.05	0.21
**Technique**	**0.17**	**0.06**	**0.05**	**0.30**

DV = Donation worth				
Effect	*b*	*SE*	95% LLCI	95% ULCI
Direct effect	0.22	0.17	− 0.10	0.55
Indirect effect				
Total	0.15	0.09	− 0.03	0.33
Emotion	− 0.14	0.11	− 0.38	0.06
**Intellect**	**0.24**	**0.09**	**0.09**	**0.44**
Novelty	− 0.12	0.08	− 0.28	0.02
Place	0.04	0.06	− 0.09	0.16
Interaction	0.04	0.09	− 0.14	0.20
Technique	0.09	0.07	− 0.03	0.25

Significant result is bold.

Regarding overall satisfaction, the direct effect was nonsignificant, while the total indirect effect of SAE was significant. Specifically, individual factors of emotion and technique had significant indirect effects.

Regarding donation worth, the direct effect was nonsignificant, and the total indirect effect of SAE was also nonsignificant. However, individual factor of intellect had a significant indirect effect.

In sum, SAE fully mediated the effect of street performance on overall satisfaction, and SAE factor of intellect fully mediated the effect of street performance on donation worth. Thus, H6a and H6b were both supported.

## Discussion

To verify the effect of street performance on perception of public space and examine the mediational role of SAE in this effect, a between-subjects field experiment was carried out in a public space in Hong Kong. The present study has yielded three main findings.

First, the perception of public space without vs. with busking did not differ significantly in terms of essentialism (H1a), anti-essentialism (H1b), nor overall liking (H1d). The presence of busking only enhanced the sonic restorativeness of the space (H1c). Within the space with busking, however, engaged audience perceived the space as significantly more essentialist (H2a), anti-essentialist (H2b), sonically restorative (H2c), and likeable (H2d) than unengaged passersby did. These results remained unchanged after controlling for frequency of visit. Thus, while H1 was partially supported, H2 was fully supported. These findings are consistent with previous findings^23^. One possible interpretation is that the effect of street performance requires the audience’s genuine attention to or engagement with the performance. The mere presence of street performance in a public space might not suffice to influence people’s perception of the space. It is among those who have stopped to pay attention to or participate in as an audience that the space is perceived significantly more favorably. Consequently, this interpretation suggests that sonic restorativeness is an exception. This exception is reasonable, given that street music represented street performance in our study, and sonic restorativeness was assessed with a focus on the soundscape of the public space. As previously mentioned, due to its sonic nature, street music can be omnipresent and permeate every corner of a public space^18,21^. Hence, the street music in our study could have been heard without being consciously noticed, and its mere presence might have been sufficient to enhance the sonic restorativeness of the space, even among those not paying attention to it. In a broader context, this finding implies that music listening can enhance the sonic restorativeness of a setting. Theoretically, these findings can be understood under the framework of place essentialism put forth by Lewicka^29^, which emphasizes that the perception of a setting (e.g., evaluations of essentialism, anti-essentialism, and overall liking) could largely depend on visual characteristics of the setting influencing human visual perception. From such a perspective, then, it is reasonable that street music appeared more of a sonic manipulation than a visual one in the present study.

Second, unsurprisingly, engaged audience experienced significantly more emotion (H3a), intellect (H3b), novelty (H3c), place (H3d), interaction (H3e), and technique (H3f) compared to unengaged passersby. This establishes a foundation for investigating SAE as a mediator of the effect of street performance. As hypothesized, SAE fully mediated the differences between unengaged passersby vs. engaged audience in their perception of public space in terms of essentialism (H4a), anti-essentialism (H4b), sonic restorativeness (H4c), and overall liking (H4d). Specifically, the SAE factor of place mediated the effects on essentialism, sonic restorativeness, and overall liking, highlighting the placemaking aspect of street performance in the context of environmental perception. These results were significant after controlling for frequency of visit, expertise, familiarity, and interest, and they were unaffected by the mood of the songs performed. Thus, engaged audience are more likely to enjoy a more intense experience of street performance, leading them to perceive the surrounding public space more favorably than unengaged passersby do. These findings not only demonstrate SAE as an explanatory framework for the effect of street performance on the perception of public space, but also provide empirical support for the argument that the effect of street performance requires audience engagement. Broadly speaking, immersing or embedding oneself in art experiences even in an uncontrollable or unpredictable setting such as the public space can lead people to positive outcomes–this is in line with Tay et al.’s^42^ conceptual model of the role of the arts and humanities in human flourishing.

Third, consistent with previous studies^13,24,25,28^, engaged audience were significantly more likely to feel satisfied with the street performance (H5a) and perceive the performance as worth donating to (H5b) compared to unengaged passersby. SAE factors of emotion and technique could account for the effect on overall satisfaction (H6a). Thus, as engaged audience experienced more emotion and technique, they also felt more satisfied with the performance. SAE factor of intellect could account for the effect on donation worth (H6b). Thus, as engaged audience experienced more intellect, they also perceived the performance as worthier of donation. These results were significant after controlling for expertise, familiarity, and interest, and they were unaffected by the mood of the songs performed. These findings are intelligible in light of Leder et al.’s^40^ model of aesthetic experience, according to which the audience’s involvement in a given aesthetic stimulus implies a subjective understanding of the stimulus, which is in turn associated with a more satisfying experience and favorable evaluation of the stimulus. Overall, these findings demonstrate the psychological consequences of SAE, and they may inform practitioners of street performance or street art in the real world.

In comparison with the previous literature on street performance^1,3,7,13,17,19,21–24^, the present study has several similarities and differences. In terms of similarities, this study validates the impact of street performance on the perception of public space, highlights the passersby’s and audience’s perspective, and adopts field research for the advantage of ecological validity. In terms of differences, this study expands the mediating effect of SAE and incorporates variables of place perception derived from environmental psychology. Either way, the present study is consistent with the previous field experiment^23^. Both the present and previous studies indicate that, while public space without vs. with busking seem to differ only in perceived restorativeness, unengaged passersby vs. engaged audience tend to differ significantly in all variables of public space perception. Hence, these studies agree that the mere presence of (musical) street performance could be insufficient to influence public space perception, and that genuine audience engagement is crucial for street performance to exert a positive impact.

Our study has certain limitations. First, we employed a field experiment for the research design. As a quasi-experiment, the participants were not randomly assigned, and their groups or conditions might simply reflect some preexisting characteristics such as expertise, familiarity, and interest in relation to street performance. While we controlled for potential confounds and found significant results throughout the analyses, future studies may consider research designs where random assignment is feasible to eliminate self-selection effects. For example, a laboratory setting where street performance may be presented in the form of video or virtual reality can strike a balance between ecological validity and experimental control. Second, our study mostly represents a situation where local people encounter street performance in a local area, which differs from situations where tourists encounter street performance in a tourist area^11,43,44^. While local people’s expectations about a local area might be more mundane and utilitarian, tourists might be more likely to actively seek, receive, join in, and appreciate surprising events as they wander through a tourist area. Future studies may focus on street performances in tourist areas. Third, our study may be limited to the particular location where the field experiment took place. Different types of public space serve different purposes^9^. For example, while some spaces primarily serve the purpose of commuting (e.g., squares and streets), others primarily serve the purpose of rest (e.g., parks and waterfronts)^45^. Our findings may not generalize to all types of public space. Future studies conducted in various public spaces worldwide should consider the character of a place when examining street performance. Fourth, our study focused on the musical type of street performance and so our findings may not generalize to the nonmusical type. Musical busking can be listened to from a distance or as people pass by, whereas nonmusical busking entails gathering around the performance to watch it happen. Hence, different types of street performance may lead to different expectations about the public space or street performance under question. Future studies should explore the potential moderating impact of performance type in the effect of street performance or SAE. Fifth, our study did not control for perceived congestion or crowding of the public space. Perceived congestion might influence participants’ overall perception of the space. For example, perceived crowding might influence the perceived suitability of the space for street performance and thereby influence SAE^24^. Future studies should consider this potential confounding variable.

In closing, the present work may have practical implications. The legitimacy of street performance has always been controversial. “Much of the history of street performance… is found in laws that prohibit it” (p. 22), as Harrison-Pepper^7^ summarizes. While it has been argued that street performance should be seen as a fundamental right to free expression^46^, various regulations are imposed on it in cities around the world, with some being acknowledged as potentially conducive^2,3,39^ and others criticized as ineffective^16,20,47^ to the busking culture. As Astor^43^ points out, the status of street performers and their freedom to express in the public space depend critically on whether their presence is perceived as a desirable or undesirable element of urban life. Also, as shown in Green’s^47^ interviews with government representatives, regulations of street performance from a policymaker’s perspective are typically concerned with managing the order of a city. Thus, favorable or unfavorable views on street performance may have real consequences in the regulations of street performance. The present work provides empirical support for the positive impact of street performance in the public space. This is largely in line with the recent research indicating the benefits of artistic interventions on the well-being of city inhabitants^48^. Hence, the relationship between street performance, as an artistic intervention, and the life quality of city inhabitants should inform policymakers.

### Supplementary Information

Supplementary Information.

## Data Availability

The data sets generated and analyzed during the current study are available in the *Open Science Framework* repository: https://osf.io/j52ys.
