# Supplementary material for: Field experiment on the effect of musical street performance/busking on public space perception as mediated by street audience experience
Source: Sci Rep. 2024 Jun 7;14:13147. doi: 10.1038/s41598-024-62672-1 (PMC11161492; doi:10.1038/s41598-024-62672-1)
Supplement: Supplementary file 1 — Supplementary Information. [file 41598_2024_62672_MOESM1_ESM.pdf]

# SUPPLEMENTARY INFORMATION

## Supplementary method

### Pretest on mood of songs

For variety of songs, the busker was asked to prepare two sets of songs, one intended to convey a positive/happy mood and the other one negative/sad. The busker proposed eight positive songs and six negative songs. All songs were pretested to ensure that the intended moods were aligned with the perceived moods. With a within-subjects design, complete recordings of the original songs were assessed by 55 (59.8%) female and 37 male (40.2%) listeners aged 19–40 years ($M$ = 23.6, $SD$ = 4.2). Each recording was rated in terms of its perceived positive mood and negative mood on a 7-point scale (from 1 = *not at all* to 7 = *extremely*). Results are summarized in Table S1. All positively intended songs were more likely to be perceived as positive; all of them were simultaneously significantly above the midpoint of the positive scale and below the midpoint of the negative scale. All negatively intended songs were more likely to be perceived as negative; all of them were simultaneously significantly below the midpoint of the positive scale and above the midpoint of the negative scale.

## Supplementary table

Table S1

*Descriptive statistics and one-sample* t *tests on the perceived moods of the selected songs*

| Song | Perceived positive mood | | | | | Perceived negative mood | | | | |
|---|---|---|---|---|---|---|---|---|---|---|
| | *M* | *SD* | *M* – 4 | *t*(91) | *p* | *M* | *SD* | *M* – 4 | *t*(91) | *p* |
| Intended positive mood | | | | | | | | | | |
| Happy Ferris Wheel | 4.97 | 1.56 | 0.97 | 5.96 | .000 | 2.46 | 1.49 | –1.54 | –9.97 | .000 |
| Needing You Every Minute | 5.87 | 1.36 | 1.87 | 13.18 | .000 | 1.72 | 1.07 | –2.28 | –20.42 | .000 |
| So I Say I Love You | 5.75 | 1.28 | 1.75 | 13.11 | .000 | 1.88 | 1.35 | –2.12 | –15.07 | .000 |
| Life is Priceless | 6.51 | 0.98 | 2.51 | 24.63 | .000 | 1.39 | 0.98 | –2.61 | –25.46 | .000 |
| Momoko Sakura's Mind | 5.60 | 1.54 | 1.60 | 9.95 | .000 | 1.77 | 1.16 | –2.23 | –18.44 | .000 |
| Magic Guru Guru | 6.14 | 1.14 | 2.14 | 17.95 | .000 | 1.45 | 1.04 | –2.55 | –23.52 | .000 |
| Compassion 100 Times | 6.36 | 1.00 | 2.36 | 22.60 | .000 | 1.30 | 0.69 | –2.70 | –37.41 | .000 |
| Automatic Victory Let's Fight | 6.55 | 0.83 | 2.55 | 29.51 | .000 | 1.30 | 0.59 | –2.70 | –43.96 | .000 |
| Intended negative mood | | | | | | | | | | |
| Better Not to Meet | 1.98 | 1.01 | –2.02 | –19.29 | .000 | 5.53 | 1.17 | 1.53 | 12.55 | .000 |
| The Book of Laughter and Forgetting | 2.46 | 1.44 | –1.54 | –10.28 | .000 | 5.12 | 1.46 | 1.12 | 7.36 | .000 |
| Next Year Today | 2.66 | 1.43 | –1.34 | –8.96 | .000 | 5.14 | 1.55 | 1.14 | 7.05 | .000 |
| Wood Grain | 2.01 | 1.19 | –1.99 | –16.03 | .000 | 5.40 | 1.24 | 1.40 | 10.84 | .000 |
| Kite | 2.30 | 1.15 | –1.70 | –14.19 | .000 | 5.17 | 1.40 | 1.17 | 8.02 | .000 |
| Memory Foam | 2.07 | 1.23 | –1.94 | –15.09 | .000 | 5.54 | 1.31 | 1.54 | 11.28 | .000 |

# Supplementary results

## Preliminary analyses

### *Effect of participant group on frequency of visit*

A one-way ANOVA with participant group as a between-subjects factor revealed a significant difference in frequency of visit ($F(2, 286) = 8.71$, $p < .001$, partial $\eta^2 = .06$). This difference was followed up by post hoc comparisons. The control group ($M = 5.79$, $SD = 1.49$) differed significantly from both the experimental unengaged passersby ($M = 4.68$, $SD = 1.88$, $p < .001$) and engaged audience ($M = 5.19$, $SD = 1.74$, $p = .029$). Unengaged passersby did not differ significantly from engaged audience ($p = .107$).

### *Effect of participant group on expertise, familiarity, and interest*

Two-way ANOVAs with unengaged passersby vs. engaged audience and mood of songs as between-subjects factors revealed significant differences in expertise ($F(1, 193) = 4.32$, $p = .039$, partial $\eta^2 = .02$), familiarity ($F(1, 193) = 4.34$, $p = .039$, partial $\eta^2 = .02$), and interest ($F(1, 193) = 7.11$, $p = .008$, partial $\eta^2 = .04$). In comparison with unengaged passersby, engaged audience reported higher expertise ($M = 2.61$, $SD = 1.87$ vs. $M = 2.01$, $SD = 1.48$), higher familiarity ($M = 4.06$, $SD = 1.70$ vs. $M = 3.53$, $SD = 1.66$), and higher interest ($M = 5.33$, $SD = 1.36$ vs. $M = 4.73$, $SD = 1.51$). Mood of songs had no significant main effects on expertise ($F(1, 193) = 0.56$, $p = .457$, partial $\eta^2 = .00$), familiarity ($F(1, 193) = 0.02$, $p = .892$, partial $\eta^2 = .00$), nor interest ($F(1, 193) = 0.34$, $p = .558$, partial $\eta^2 = .00$). Mood of songs also had no significant interaction effects on expertise ($F(1, 193) = 0.73$, $p = .396$, partial $\eta^2 = .00$), familiarity ($F(1, 193) = 0.07$, $p = .798$, partial $\eta^2 = .00$), nor interest ($F(1, 193) = 1.15$, $p = .284$, partial $\eta^2 = .01$).
